# Unraveling the role of autophagy and antioxidants in anther and pistil responses to heat stress in rapeseed (*Brassica napus L*.)

**DOI:** 10.1007/s00299-025-03437-6

**Published:** 2025-02-07

**Authors:** Valiollah Mohammadi, Ahmad Rezaeizadeh, Behnam Mondak, Abdolrahman Rasoulnia, José Domínguez-Figueroa, Laura Carrillo, Gara Romero-Hernandez, Joaquin Medina

**Affiliations:** 1 Department of Agronomy and Plant Breeding, College of Agriculture and Natural Resources, University of Tehran, Karaj, Iran; 2 Centro de Biotecnología y Genómica de Plantas (CBGP), UPM-INIA/CSIC, Campus de Montegancedo, Madrid, Spain; 3 Facultad de Ciencias Ambientales y Bioquímica, Universidad de Castilla-La Mancha, Toledo, Spain

**Keywords:** Heat, Stress, Brassica, Anther, Pistil, Autophagy, Histochemical analysis, Superoxide dismutase (SOD), Catalase (CAT), *ATG8d*, *EXO70B*, *NBR1*

## Abstract

**Key message:**

Enhanced antioxidant enzymes activity, particularly superoxide dismutase and catalase, along with autophagy process in reproductive organs, can improve the resilience of rapeseed to heat stress, thereby securing crop yield in the face of global warming.

**Abstract:**

Climate change and global warming have increasingly influenced yield and quality of rapeseed (*Brassica napus*) almost all across the world. The response of reproductive organs to high-temperature stress was studied in two rapeseed varieties, SAFI5 and DH13 with contrasting levels of heat stress tolerance. Pollen germination, viability, and seed set showed a significant reduction in the heat-sensitive variety (DH13). Superoxide quantification revealed higher accumulation in heat-sensitive variety, leading to decreased seed formation and floret fertility most probably due to declined pollen viability and stigma receptivity. Further microscopic analysis of the anther and pistil demonstrated a significant overlay between the damaged areas and the location of O_2_^−^ accumulation. The sensitive variety showed higher O_2_^−^ accumulation and a wider damage area than the tolerant one, suggesting that superoxide could incapacitate anther and pistil due to structural injury. Moreover, the activity levels and expression of superoxide dismutase and catalase antioxidant enzymes were significantly higher in the anther and pistil of the tolerant variety. Histochemical analysis also indicated markedly higher autophagosome formation in tolerant variety’s anther and pistil. Consistently, the expression levels of *autophagy* and *ubiquitin-proteasome system (UPS)-related* genes including *BnATG8d*, *BnEXO70B*, *BnATl1 4A*, and *BnNBR1*, as well as ubiquitin-activating enzyme *E1*, were higher in both reproductive organs of the tolerant variety. Interestingly, the areas of autophagosome formation overlapped with the areas in which higher superoxide accumulation and structural changes happened, suggesting a specific role of autophagy in oxidative stress response.

**Supplementary Information:**

The online version contains supplementary material available at 10.1007/s00299-025-03437-6.

## Introduction

Heat stress (HS) is one of the major limiting factors that impact crop productivity and geographic distribution worldwide (Porter 2005; Kourani et al. 2022). This issue is projected to intensify in the coming decades due to global warming driven by elevated atmospheric carbon dioxide and extensive land transformation (Aksouh et al. 2001; Sage et al. 2015; Mohammadi et al. 2018). The observed increase in ambient temperatures by + 1.4 to + 5.8 °C during the period of 1990–2010 highlights the necessity for a thorough understanding of HS and its implications (IPCC 2022).

Rapeseed (*Brassica napus*), the second largest source of vegetable oil worldwide, is particularly sensitive to HS during flowering and pod formation as a cool season crop (Anissa et al. 2013; Gan et al. 2004; Chen et al. 2019). Previous studies indicate that seed yield can decline by 85.3% under HS (Elferjani and Soolanayakanahally 2018). Notably, as few as 3 days HS from the green bud stage to 2 weeks after the first open flower can significantly decline pod number and seed yield on the main stem (Chen et al. 2020). Furthermore, short periods of HS extending up to 5 weeks after the first open flower on the main stem continue to decrease pod number and seed yield on the branches (Chen et al. 2020).

It is well documented that plant reproductive organs are among the most sensitive physiologic components to elevated temperatures. Pollen viability decline and floret sterility increase markedly if daily temperature exceeds 38 °C at the meiosis stage, even for one day (Liu et al. 2023). In addition, exposure to high temperatures around 33 °C during the meiosis stage may lead to pollen abortion and floret sterility without affecting vegetative development (Prasad et al. 2006; Krishnan and Ramakrishnan 2011). Previous studies have reported that floral sterility, which affects both male and female reproductive organs, occurs when rapeseed plants are exposed to HS during flowering (sheng et al., 2021; Chen et al. 2021). In fact, the significant yield loss induced by HS is mostly due to pollen abortion and lack of pollination or fertilization. Similarly, several other studies have also revealed that HS causes poor anther dehiscence, leading to reduced pollen dispersal in crops like rice and tomato (Sato et al. 2002; Madan et al. 2012).

Plant response to HS varies with species, developmental stage, tissue, stress severity timing, etc. (Nejat and Mantri 2017). One of the initial effects of all environmental stresses is the generation of ROS in plants (Zhao et al. 2018). Despite the unfavorable effect of ROS, numerous studies have revealed that ROS also acts as signaling molecules regulating various biologic processes (Choudhury et al. 2013; Corpas et al 2019, 2020; Mittler 2017;). Excessive ROS generation in plants can lead to tissue-specific programmed cell death (PCD) (Karuppanapandian et al. 2011), which is closely associated with pollen sterility (Zhang et al. 2023; Wan et al. 2007). To counter the harmful effects of ROS, plant activate mechanisms such as ROS scavenging and the production of antioxidants including superoxide dismutase (SOD), peroxidase, catalase (CAT), and ascorbate peroxidase (APX) (Corpas et al. 2020; Gill and Tuteja 2010; Fortunato et al. 2023).

HS results in protein misfolding and denaturation, thereby cellular proteome integrity. These misfolded proteins and their aggregates are potentially toxic to the cell and must be refolded, degraded, or sequestered into specialized compartments. Heat shock proteins (HSPs) contribute highly to protecting the cellular proteome after HS by refolding the unfolded proteins or supporting their degradation either alone or in collaboration with other degradation pathways such as the ubiquitin–proteasome pathway (Wang et al., 2004; Chen et al., 2011). The protein ubiquitin–proteasome system *(UPS)* is a multistep reaction mediated by three enzymes including E1 (ubiquitin-activating enzyme), E2 (ubiquitin-conjugating enzyme), and E3 (Ubiquitin-ligase enzyme).

Autophagy meaning ‘self-eating’ is another pathway involved in protecting proteins under stress through bulk degradation of cytoplasmic materials by vacuole or lysosome in eukaryotes (Marshall and Vierstra 2018; Petersen et al. 2024). This will also help cells to maintain energy balance. Several studies have reported the significant contribution of autophagy to HS response (Lohani et al. 2024; Li et al 2023; Zhou et al. 2013; Wang et al. 2016; Dundar et al., 2019; Sedaghatmehr et al. 2019; Bárány et al., 2018; Su et al. 2020). Outrageous accumulation of aggregated proteins as well as lower heat tolerance after declined autophagy in Arabidopsis and tomato highlight the significant role of this mechanism in removing toxic and aggregated proteins under HS (Zhou et al. 2013, 2014).

In previous work, we evaluated a panel of commercial and promising spring rapeseed varieties for HS tolerance during the flowering stage under greenhouse conditions (Mohammadi et al. 2018). This study revealed significant variation in heat tolerance with respect to plant seed yield. Notably, the DH13 cultivar was identified as the most heat-sensitive, showing a substantial reduction in grain yield by 47%, whereas SAFI was the most tolerant one. Since there is a close correlation between yield loss induced by HS and pollen abortion and lack of pollination or fertilization, we decided to investigate the specific responses of reproductive organs to high-temperature stress in two rapeseed varieties, SAFI5 and DH13. In the current study, we conducted a more detailed analysis of the specific effects of short-term HS on male and female reproductive organs and investigated molecular mechanisms underlying heat tolerance in these contrasting varieties. Our work provides a theoretical framework for enhancing HS resistance in *Brassica napus*, emphasizing the crucial roles of both antioxidant enzyme activities and autophagy processes. As little is known about the mechanism of heat tolerance in rapeseed reproductive organs, these findings would offer valuable insights into physiology of and breeding for heat tolerance, which is vital for ensuring stable and sustainable crop production in the face of global climate change.

## Material and methods

### Plant materials and heat stress treatment

Two rapeseed varieties with contrasting HS tolerance, SAFI5 (tolerant) and DH13 (sensitive) were selected for this study based on our previous screening of a collection of rapeseed varieties under different HS conditions. HS applied by a plastic greenhouse at flowering stage equipped with electric heater. Based on grain yield in normal and heat-stressed condition and tolerance indices (SI, SSI, STI, TOL and MP), SAFI5 and DH13 were shown to be the most tolerant and susceptible varieties with 47 and 20% yield loss, respectively (Mohammadi et al. 2018).

For the heat-stress treatments, seeds of the two varieties were germinated in 20-cm diameter pots and raised in a growth chamber with 22/18 °C, 16/8 h, photoperiod. Plants were randomly divided into stress and control groups at the flowering stage and stress ones were subjected to a high temperature of 35/22 °C (day/night) for 72 h in the growth chamber. After heat-stress treatment, anthers and pistils of plants were collected immediately and stored at -80 °C for further analysis.

### Pollen germination and viability test

Pollen viability was evaluated by the Evans Blue staining method described by Barany et al. (2018). Samples were incubated with a 0.25% (*w*/*v*) aqueous solution of Evans Blue for 30 min and observed with a light microscope under a bright field. The mean percentage of viable pollen (Non-stained by Evans Blue) was quantified in random micrographs.

For germination analysis, fresh harvested pollens were spread on a medium consisting of 200 g sucrose (C_12_H_12_O_11_), 10 mg of boric acid (H_3_BO_4_), 63 mg of calcium chloride (CaCl_2_), 20 mg of potassium nitrate (KNO_3_) and 40 mg of TRIS dissolved in 1 L of de-ionized water and stored in dark room temperature. A fresh solid germination medium was prepared for both temperature treatments by adding 1% agar to the appropriate amount of stock medium. Once the agar was completely dissolved, 10 ml of the medium was poured into each of the four replicated Petri dishes for each variety and each treatment. After the agar solidified, the Petri dishes were covered and incubated at 20 °C and 35 °C temperatures, and germination percentages were measured after 48 h. The length of 100 pollen tubes was measured by an ocular micrometer fitted into the eyepiece of the microscope and converted into micrometer units after calibration with a stage micrometer. For both rapeseed varieties, the number of seeds per pod was monitored. Each pod was opened and evaluated at physiologic maturity. An aborted ovule was determined that failed to develop into a seed, whether it had been fertilized or remained unfertilized (Singh et al. 2008).

### Enzyme extraction and assay

Frozen pistils and anthers (50 mg) were ground into a fine powder and homogenized in 1000-μL 50-mmol Tris–HCl (pH 7.8) containing 1% PVP (*w*/*v*), 1-mmol PMSF, 0.1% Triton-X100 (*w*/*v*) and 0.1-mmol EDTA. The homogenate was centrifuged at 13,000 × g for 15 min at 4 °C, and the supernatant was dispersed in aliquots and stored at − 80 °C until further analysis (Ozgur et al. 2014). Based on the method developed by Beauchamp and Fridovich (1971), the activity of superoxide dismutase (SOD) was measured spectrophotometrically. Ascorbate peroxidase (APX) activity was determined by the decrease of absorbance at 290 nm as described by Chen and Asada (1989). Catalase (CAT) activity was determined at 240 nm by its ability to the reduction of H2O2 according to the method described by Aebi (1984).

### Quantitative RT-PCR

Total RNA was extracted from pistils and anthers of rapeseed varieties subjected to high temperature (35/22 °C, day/night) and control (22/18 °C, day/night) conditions for 72 h in the growth chamber, using LiCl protocol (Oñate-Sánchez and Vicente-Carbajosa 2008). Three biological and three technical replications were taken for qRT-PCR analysis. The cDNA was synthesized from 2 µg of isolated total RNA by incubation of 5 min at 70 °C with 1 µL (1 µg) Oligo dT18-primer (Promega, Madison, WI, USA) and RNase-free dH_2_O in a total reaction volume of 13.5 µL. Then, 12.5 µL of a mix containing 2.5-mM dNTPs, 40-mM RNAsin, 200 U of Moloney Murine Leukemia Virus (M-MLV) reverse transcriptase, and 1 µM of DTT were added. The solution incubated for 1 h at 37 °C and subsequently diluted to 1:1 with dH2O. Samples were incubated for another 5 min at 80 °C and stored at − 20 °C until further processing. QuantPrime was used to design specific forward and reverse primers. Primers and LightCycler®480 SYBR Green Master (Roche) were transferred to 96 multi-well plates according to the manufacturer’s protocol using 3.5 µL of the samples for a 25-µL reaction volume. The following conditions were applied for qRT-PCR: 10 min at 95 °C, 45 cycles of 15 s at 95 °C, 20 s at 60 °C, and 5 min at 72 °C. A third step for dissociation of 5 s at 95 °C, 1 min at 65 °C, continuous at 97 °C, and 30 s at 40 °C for melting dynamics analysis. The BnActin7 gene (*BnaA03g55890D*) was used as a reference for data normalization (Zhou et al., 2017). Relative expression levels of target genes were calculated by the 2 − ΔΔCT method (Pfaffl 2001) where ΔCt is the difference in threshold cycle number (Ct) for target genes and reference genes. The expression of *BnSOD*, *BnAPX*, *BnCAT*, *BnATG8D*, *BnEXO70B*, *BnNBR1*, and *BnATI1A* genes were evaluated. Primers used for expression analysis are shown in Table 1.

**Table 1:** Primers used for qR-TPCR expression analyses

Sequence	Gene
*BnActin7*	TGGGTTTGCTGGTGACGAT—Forward
	TGCCTAGGACGACCAACAATACT—Reverse
*BnCAT1*	CCTCTTTGACTGCTGGAACC—Forward
	TAGCACCTCTGGCATGAACA- Reverse
*BnSOD*	GTTCTGGCTGGGTTTGGC- Forward
	GTGTTTATGTACTTTCCCCTCTCG- Reverse
*BnAPX*	ACAGACACAATCCCCTTGGTG- Forward
	AGGTGTAATGCTGCCCATCC—Reverse
*BnATG8D*	CGATATCCCAATTCGACGTT—Forward
	TCGGGAGGATGCTCTTGCTT—Reverse
*BnEXO70B*	GCTCCAGCAAGCTATGTTCC—Forward
	ATCGTCTCCTTCCACCTCCT—Reverse
*BnNBR1*	CAGGAAATGGGGTTCTCTGA—Forward
	GACGATGCTAGCGACACAAA—Reverse
*BnATI1*	GACCACTTTGTATTCCCACCT—Forward
	CCCTAATCTTCCAGCATAAA—Reverse

### Histochemical detection and measurement of O_***2***_- content

The localization of superoxide accumulation was visually detected in pistils and anthers of plants using a histologic nitroblue tetrazolium (NBT) assay according to Frahry and Schopfer (2001). The samples were immersed in Na-citrate buffer (pH 6.0) containing 6-mm nitroblue tetrazolium (NBT) under light at 25 °C for 30 min and then photographed under a binocular microscope. Dark blue coloration indicates the presence of soluble formazan compound, formed when nitroblue tetrazolium (NBT) reacts with superoxide. The content of O_2_- was also measured via nitroblue tetrazolium (NBT) based on Frahry and Schopfer (2001). To gain further insight into the impact of HS on the reproductive organs, a microscopic analysis was conducted using the same experimental condition. A microscopic longitudinal profile of anther and pistil stained with toluidine blue was developed and superoxide anion was measured according to Zhao et al (2018).

### Fluorescence microscopy for visualization of MDC-stained autophagosomes

To run light microscopy, in vitro samples were taken and fixed in 4% paraformaldehyde in phosphate-buffered saline (PBS) with pH 6.8 and kept overnight at 4 ℃. Dehydrated in acetone, samples were lodged in Technovit 8100 resin (Kulzer, Germany) at 4ºC. For structural analysis, semi-thin resin sections were stained with toluidine blue and observed under a bright field. Resin sections were also kept at 4 ℃ for immunofluorescence. The monodansylcadaverine (MDC) staining method (Contento et al. 2005) was applied for the in vivo labeling of autophagic vacuoles. Fixed samples were stained with 0.05 mM of monodansylcadaverine (MDC) (Sigma-Aldrich) at room temperature for 30 min in darkness. Incubated samples were washed twice with PBS and immediately watched by fluorescence microscope. 405 and 435–483 nm were used as excitation and emission wavelengths for monodansylcadaverine (MDC), respectively (Solis et al., 2016).

## Results

### Effect of heat stress on pollen germination, viability, and seed set in contrasting rapeseed varieties

**Fig. 1:**
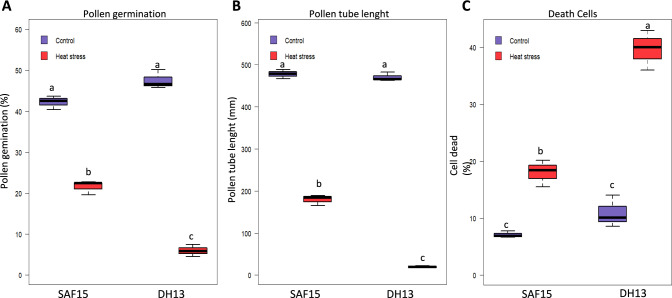
Analysis of heat stress impact on pollen germination, viability, and pollen tube growth in rapeseed varieties. Pollen germination (**A**), pollen tube length (**B**) and pollen viability (**C**) in SAFI5 and DH13 rapeseed varieties grown under non-stress (22/18 ℃) or heat-stress conditions (35/22 ℃) for three days at flowering stage. Data are means ± SD of three independent experiments. Data are means ± SE (n = 3). Letters indicate significant differences between genotypes and treatments; *p* < 0.05; Anova Student–Newman–Keuls tests. The means were compared by the least significant difference (LSD) test (p < 0.05)

To investigate the impact of HS on rapeseed male reproductive organ, we first analyzed pollen germination and viability in rapeseed varieties, DH13 and SAFI5, subjected to 35 /22ºC (heat stress) and 22/18 ℃ (as control) for three days at flowering stage. Pollen germination (%) was significantly reduced from 46.8 to 6.5% in HS sensitive variety (DH13), while it was not markedly changed in tolerant variety (SAFI5) of which pollen germination altered from 41.6 to 22.2 (Fig. 1).

**Fig. 2:**
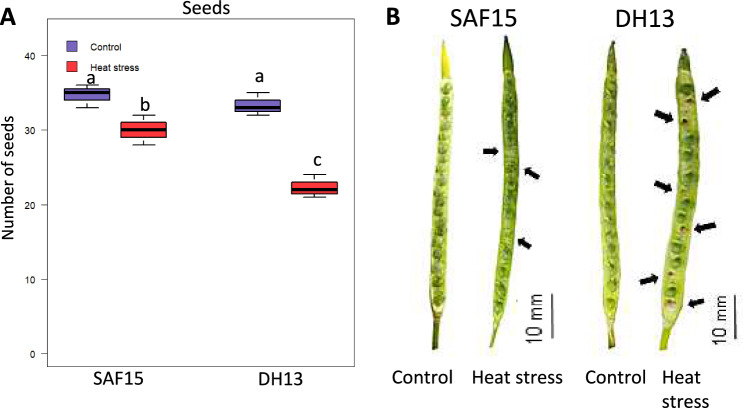
Analysis of seed number and seed formation in rapeseed varieties under heat stress. Effect of HS on seed number (**A**) and seed formation (**B**) in rapeseed varieties, SAFI5 and DH13, grown under non-stress (22/18 ℃) or heat-stress conditions (35/22 ℃) for three days at flowering stage. Letters indicate significant differences between genotypes and treatments; *p* < 0.05; Anova Student–Newman–Keuls tests. Arrows show the unfertilized and aborted seeds. Bars represent 10 mm

Pollen viability, as an important characteristic related to HS resilience, was evaluated by Evans blue staining. The results indicated that SAFI5 had significantly higher pollen viability compared to DH13 (Fig. 1). The percentage of dead pollens increased up to 11% and 28% in SAFI5 and DH13, respectively under HS. Significant differences were observed in pollen tube length and seed number between the tolerant and sensitive varieties. Notably, pollen tube lengths measured 166.7 ± 17.74 µm in tolerant variety (SAFI5) while 26.6 ± 1.99 µm in the sensitive one (DH13). Seed set decreased by 36.5% in DH13 and by only 6.7% in SAFI5 under HS (Fig. 2).

### Antioxidant enzyme activity and gene expression in anther and pistils of contrasting rapeseed varieties under heat stress

**Fig. 3:**
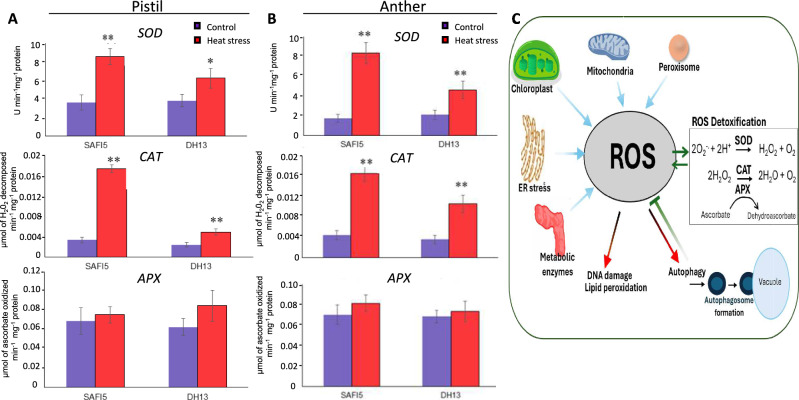
Analysis of activity of superoxide dismutase (SOD), catalase (CAT) and ascorbate peroxidase (APX) enzymes in pistil and anther of rapeseed varieties under heat stress. Activity of SOD, CAT and APX were analyzed using pistils (**A**) and anthers (**B**) extracts of rapeseed varieties, SAFI5 and DH13, subjected to 22/18 ℃ (control, blue) and 35/22 ℃ (heat stress, red) for three days at flowering stage. **C** Schematic representation of oxidative stress induced by intracellular and/or extracellular stimuli. Intracellular ROS are generated mainly in the mitochondria, as well as other organelles such as the peroxisome and endoplasmic reticulum. Concerted action of antioxidant enzymes superoxide dismutase, catalase and peroxidase mitigate oxidative stress. Data means ± SE of three independent experiments. Asterisks indicate significant differences between stressed and non-stressed, within each variety, assessed by Student’s t-test, at *p* < 0.05 (*) and *p* < 0.01 (**)

It is well established that plant cells employ various mechanisms to control ROS, including ROS scavenging and the production of antioxidants such as superoxide dismutase (SOD), peroxidase, catalase (CAT), and ascorbate peroxidase (APX) (Corpas et al. 2019; 2020; Gill and Tuteja 2010; Fortunato et al. 2023). To assess the activities of superoxide dismutase (SOD), catalase (CAT), and ascorbate peroxidase (APX) in anthers and pistils under HS, both sensitive and tolerant varieties were exposed to high temperature of 35/22 ℃ and control condition of 22/18ºC for 3 days during the flowering stage. As shown in Fig. 3, the enzymatic activities of superoxide dismutase (SOD) and catalase (CAT) in the pistil and anther were significantly higher in the tolerant variety (SAFI5) compared to the sensitive variety (DH13). However, there was no significant difference in ascorbate peroxidase (APX) activity.

**Fig. 4:**
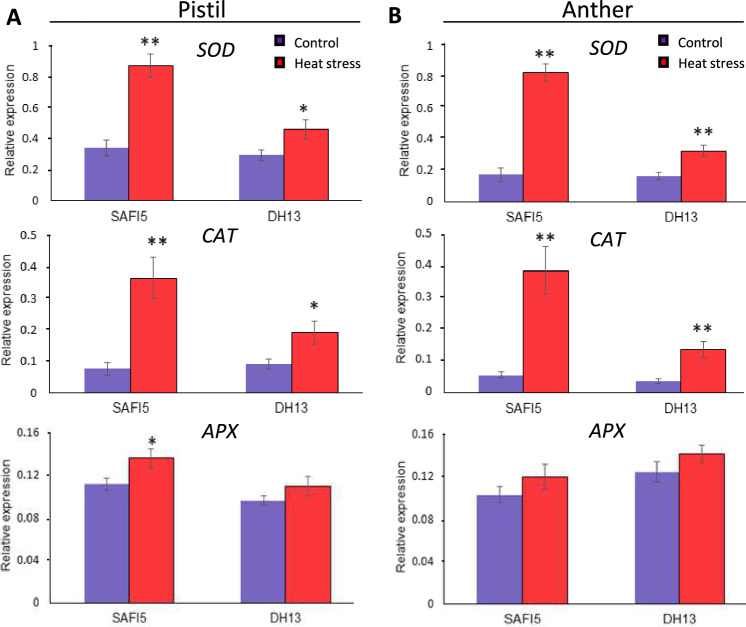
Expression analysis of of *superoxide dismutase (SOD), catalase (CAT) and ascorbate peroxidase (APX)* genes in pistil and anther in HS contrasting rapeseed varieties under heat stress. qRT-PCR expression analysis of *SOD*, *CAT* and *APX* rapeseed genes in pistils and anthers of rapeseed varieties, SAFI5 and DH13. Total RNA was extracted from pistils (**A**) and anthers (**B**) of both varieties subjected to 22/18 ℃ (as control, blue color) and 35 /22 ℃ (heat stress, red color) for three days at flowering stage. The *BnActin7* gene was used as a housekeeping gene for data normalization. Data means ± SE (n = 3). Asterisks indicate significant differences between control and stress treatment of each variety, assessed by Student’s t-test, *p* < 0.05 (*) and *p* < 0.01 (**)

To further validate these findings, we assessed the expression levels of the *BnSOD*, *BnCAT*, and *BnAPX* genes in the pistil and anther of both varieties exposed to HS. The results revealed that gene expression patterns mirrored those of the enzyme activities (Fig. 4). Specifically, the expression of the *BnSOD* and *BnCAT* genes was higher in the tolerant variety under HS, while ascorbate peroxidase (APX) gene expression was slightly elevated in the sensitive variety, although this difference was not statistically significant. These findings suggest that the production of antioxidants such as superoxide dismutase (SOD) and catalase (CAT) in the anthers and pistils may play a crucial role in conferring heat tolerance in rapeseed reproductive organs.

### Analysis of superoxide accumulation in anther and pistil of rapeseed varieties

Previous studies reported that excessive ROS generation in plants can lead to tissue-specific PCD (Karuppanapandian et al. 2011), which is usually associated with pollen sterility (Zhang et al. 2023; Wan et al. 2007). To counter harmful ROS, plants launch some mechanisms including ROS scavenging and production of antioxidants. To further support this idea, superoxide (O_2_^−^) production was estimated by nitroblue tetrazolium (NBT) staining in reproductive organs in rapeseed varieties under control and HS conditions as described previously. The level of O_2_^−^ under heat stress (HS) was higher in the heat-sensitive variety (DH13) than the tolerant one (SAFI5) and notably O_2_^−^ accumulated at the bottom of the anther near the filament (Supplementary information). In the same way, HS promoted higher levels of O_2_^−^ in pistils of sensitive variety than heat-tolerant ones. The accumulation of O_2_^−^ was mainly observed in stigma and styles. In DH13 as a sensitive variety, the whole stigma and style were fully stained indicating aggregation of O_2_^−^. Accordingly, superoxide content was higher in both organs in the heat-sensitive line than heat-tolerant line. These results supported the idea that HS accelerated O_2_^−^ production, visualized by nitroblue tetrazolium (NBT) staining, in reproductive organs. Being higher in the sensitive variety, O_2_^−^ accumulated at the bottom of anther near to filament.

**Fig. 5:**
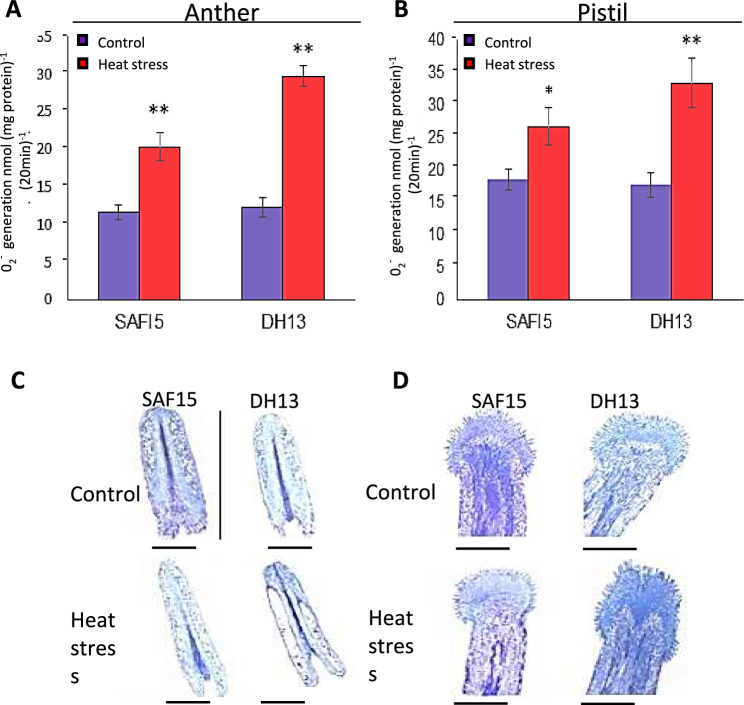
Analysis of O_2_^−^ content and accumulation in anther and pistil in contrasting rapeseed varieties. O_2_^−^ content in anther (**A**) and pistil (**B**). Microscopic analyses of longitudinal sections of anther (**C**) and pistil (**D**) stained with toluidine blue of rapeseed varieties, SAFI5 and DH13, under non-stress and heat stress conditions. Asterisks indicate significant differences between non-stress and stress conditions of each variety, assessed by Student’s t-test, at *p* < 0.05 (*) and* p* < 0.01 (**). Bars represent 1 mm

The microscopic analysis of longitudinal sections of anther and pistil stained with toluidine blue (Fig. 5 C, D) demonstrated a significant overlay between the damaged areas and the location of O_2_^−^accumulation. In anthers, the epidermis and several internal cell layers were severely affected by HS. These effects were more evident at the bottom of the anther, where O_2_^−^ accumulated. The external cell layers of the anther such as epidermis and middle layers disappeared under HS conditions, being more at the bottom of the anther, where O_2_^−^ accumulated. In pistil, most changes were observed on stigma, where O_2_^−^accumulated too. Notably, the sensitive variety (DH13) showed higher O_2_^−^ accumulation and a wider damage area than the tolerant one (SAFI5). Overall, these results suggest a close correlation between O_2_^−^ accumulation, structural damages, and seed set.

### Expression analyses of autophagy and ubiquitin–proteasome system (UPS) genes and autophagosome formation in response to HS in rapeseed varieties

Autophagy and the UPS are the two main pathways involved in the degradation of misfolded or non-functional proteins in plants (Balchin, 2016). These pathways also selectively remove regulatory proteins (e.g., developmental or stress-induced proteins) when they are not necessary anymore.

**Fig. 6:**
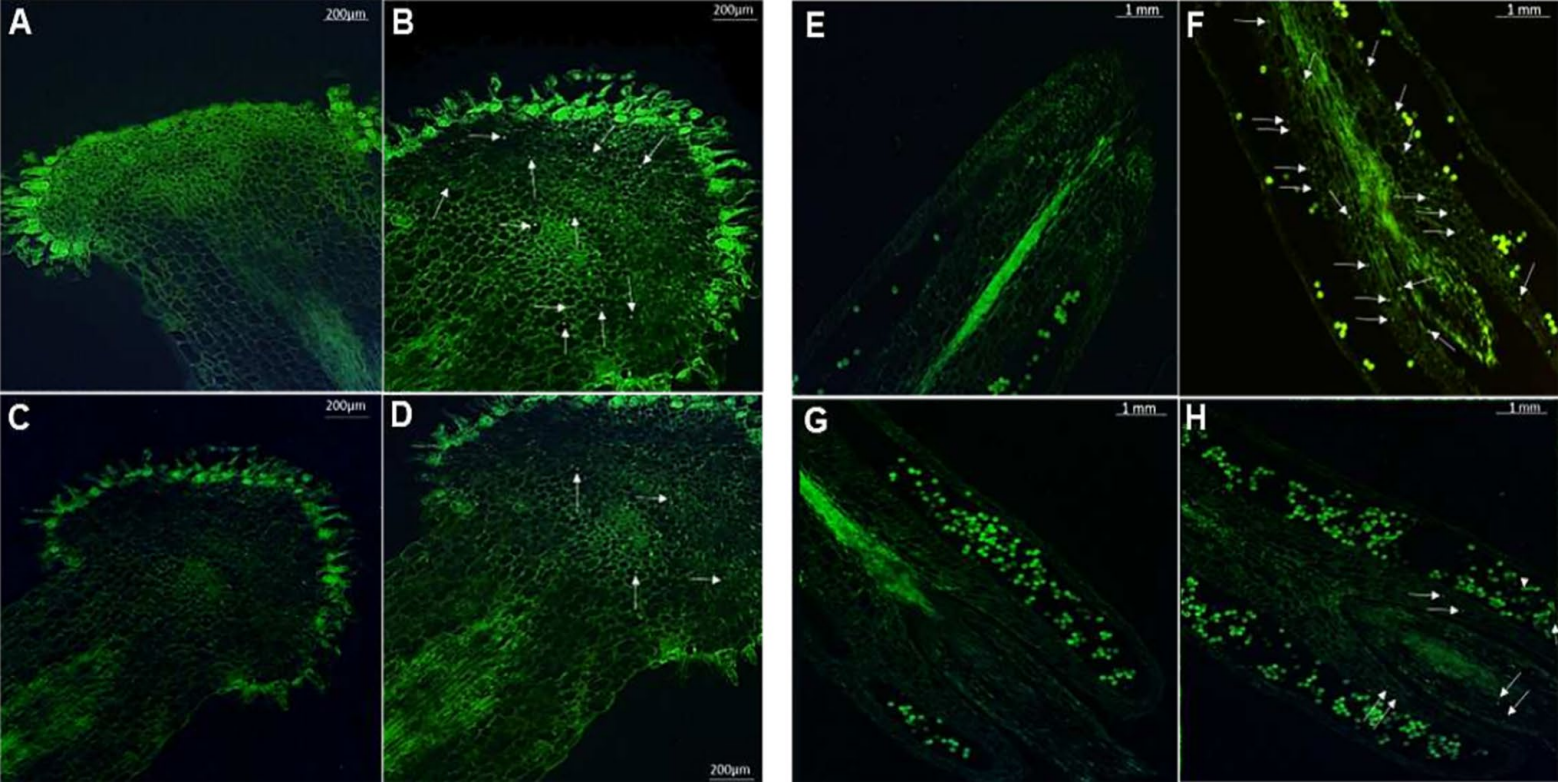
Effect of heat stress on autophagosome formation in anthers and pistils of rapeseed varieties. Fluorescence microscopy images of anthers (A, B, C, D) and pistils (E, F, G, H) of SAFI5 and DH3 varieties under non-stress (A, C, E, G) and heat-stress (B, D, F, H) conditions. Monodansylcadaverine (MDC) staining method (Contento et al. 2005) was used for in vivo labeling of autophagic vacuoles in anthers and pistils. White arrows indicate Autophagosomes. Scale bars are indicated in the picture

**Fig. 7:**
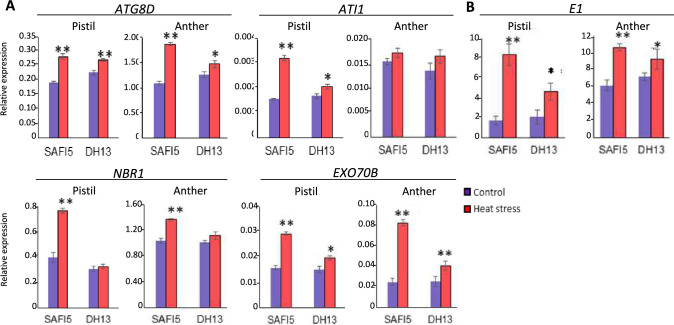
Expression analysis of *autophagy* and *UPS* genes *ATG8D, EXO70B, NBR1, ATI1* and *E1* in pistil and anther of SAFI5 and DH13 rapeseed varieties under heat stress. qRT-PCR expression analysis of autophagy of *ATG8D, EXO70B, NBR1 and ATI1* (**A**) *and UPS E1 gene* (**B**) in pistils and anthers of rapeseed varieties, SAFI5 and DH13.Total RNA was extracted from pistils and anthers of rapeseed varieties, SAFI5 and DH13, subjected to 22/18 ℃ (as non-stress) and 35/22 ℃ (as heat-stress) for three days at flowering stage. The *BnActin7* gene was used as a housekeeping gene for data normalization and the expression level was measured by qRT-PCR. Data means SE (n = 3). Asterisks indicate significant differences between non-stress and stress treatments within each variety, assessed by Student’s t-test, *p* < 0.05 (*) and *p* < 0.01 (**)

To investigate the role of both pathways in the responses to HS, we analyzed the expression levels of key genes in UPS like ubiquitin-activating enzyme *E1*, and autophagy like *BnATG8d*, *BnEXO70B*, *BnATI1*, and *BnNBR1* genes in pistils and anthers of both contrasting rapeseed varieties subjected to 22/18 ℃ (as control) and 35/22 ℃ (heat stressed) for 3 days at flowering stage. The results indicated that the expression level of in UPS like ubiquitin-activating enzyme *E1* gene was significantly different in both varieties under HS compared to the control. Notably, the expression levels of the *E1* gene under HS were higher in the tolerant variety than in the sensitive one both in pistil and anther (Fig. 7). In line with these data, the autophagy-related genes *BnATG8d*, *BnEXO70B*, *BnATl1* and *BnNBR1* showed higher levels of expression after HS treatment in the tolerant one. The expression levels of most genes were higher in the pistil and anther of the tolerant variety than the sensitive one. On the contrary, the expression level of *BnATl1* gene was slightly higher in the pistils of the sensitive variety. All these results might suggest that HS activates the autophagy process at higher levels in the heat-tolerant cultivar than in the sensitive one. To further confirm this point, a histochemical analysis was conducted by monodansylcadaverine (MDC) staining of pistils and anthers to visualize autophagosome formation. Light microscopy revealed a higher number of autophagosomes in both organs of both varieties after HS treatment, most of the autophagosomes being localized at the top of pistils and bottom of anthers (Fig. 6). The number of autophagosomes in SAFI5 (HS-tolerant), however, was higher than DH13. Interestingly, the areas of autophagosome formation overlapped with the areas in which higher superoxide accumulation and structural changes were made (Figs. 5 and 6), suggesting a specific role of autophagy in oxidative stress responses in the HS tolerant variety, SAFI5.

## Discussion

Rapeseed* (Brassica napus)* is a key oilseed crop playing a crucial role in global agriculture. Its seeds are rich in oil and protein, making it an important source for oil, animal feed and biodiesel. Containing unsaturated fatty acids, omega-3 and omega-6 acids, fat-soluble vitamins, and lack of cholesterol has turned rapeseed oil to a high-quality oil for regular consumption. As climate change increasingly impacts agricultural systems worldwide, it has become essential to deeply understand heat tolerance mechanism of the plants to ensure sustainable crop production. In our current study, we focused on two specific rapeseed varieties, SAFI5 and DH13, known for their distinct responses to HS. This research was aimed to provide insights into the molecular mechanisms at play and to determine how our findings may be applicable to other varieties or different environmental contexts. In our earlier research (Mohammadi et al. 2018), a diverse selection of commercial and promising spring rapeseed varieties was assessed for HS tolerance during flowering stage. This study revealed considerable genetic variation in heat tolerance among the varieties tested, particularly in terms of seed yield. DH13 variety was identified as highly sensitive to HS, experiencing a significant 47% decrease in grain yield, while SAFI5 showed greater resilience with only a 20% reduction in yield under similar condition. These findings also highlight the importance of selecting appropriate cultivars based on their heat tolerance characteristics, especially as temperatures continue to rise due to climate change.

The impact of HS on plant yield varies with several factors, including species, genotype, developmental stage, and the duration, intensity, and rate of temperature increase (Fábián et al. 2019). HS during the reproductive phase can significantly restrict yield due to the heightened sensitivity of pollen and pistils (Sage et al. 2015; Chen et al. 2020; Macova et al. 2022). Our data demonstrated that HS resulted in reduced pollen viability, shorter pollen tube lengths, and lower seed set (Figs. 1 and 2), with more severe damage observed in the susceptible variety compared to the tolerant one (Figs. 2 and 5). These results are in line with previous research (Jagadish et al. 2007; Fábián et al. 2019; Djanaguiraman et al. 2018; Zhao et al. 2018). HS influences nearly all reproductive processes in plants, including meiosis, the quantity and viability of pollen and ovules, pollen germination and movement within the stigma, fertilization, embryo development, and seed formation (Sakata et al. 2000; Chen et al. 2021). Abnormal ovule development under HS has been reported, while reduced pollen viability has been documented by Chen et al. (2020). Some studies indicate that male reproductive organs are more vulnerable to HS than female organs (Endo et al. 2009; Giorno et al. 2013; Sage et al. 2015), although others argue that female organs are equally susceptible (Chen et al. 2021; Macova et al. 2022). In terms of pollen viability and floret fertility, Zhao et al. (2018) suggested that the level of HS-induced damage would increase with rising temperatures and prolonged exposure.

This study also found that seed formation in both varieties was severely impacted by short-term HS conditions, but the sensitive variety showed more damage than the tolerant, as expected (Fig. 5). These results align with those reported by Macova et al. (2022) and Chen et al. (2020) who observed reduced viable seed yield under long-term HS in rapeseed. This reduction in seed yield was attributed to factors such as decreased fertilization, increased abortion, incomplete development of embryos, and preharvest sprouting. Consistently, Elferjani and Soolanayakanahally (2018), also reported a reduction in seed yield (85.3%) under long-term HS and a lesser extent under drought conditions (31%), emphasizing the significant effect of high temperature on seed formation in rapeseed.

Environmental stresses in plants often lead to secondary stress known as oxidative stress arising from upsurged ROS. It is well established that HS causes a significant increase in O_2_^−^ accumulation in the developing reproductive organs of plants (Frank et al. 2009; Zhao et al. 2018). In situ detection of O_2_^−^ concentration in anther and pistil provided sufficient evidence that HS significantly enhances ROS accumulation (Fig. 5 A–F). Aligning O_2_^−^ content (Fig. 5 A, B) and accumulation (Fig. 5 C–F) with pollen attributes under HS (Fig. 1), implies that O_2_^−^ accumulation has likely led to decreased pollen viability and seed set. According to Zhao et al. (2018), ROS levels seriously increased in rice anthers in line with the intensity and duration of HS exposure, associated with lower pollen viability and higher floret sterility. Our data strongly reinforced several earlier findings that HS raised ROS accumulation in reproductive organs as documented in tomato and rice anthers (Frank et al. 2009; Zhao et al. 2018). Moreover, previous studies reported that ROS accumulation in plants triggers lipid peroxidation in cell membranes resulting in lower membrane thermostability membranes (Hasanuzzaman et al. 2014), as well as aberrant PCD during anther development (Wan et al. 2007). It was found in our study that the susceptible rapeseed variety showed greater ROS concentration in the anther (Fig. 5) suggesting that HS-induced ROS accumulation in developing anther was most likely responsible for declined pollen viability and increased floret sterility (Gill and Tuteja 2010). Overall, these observations further support the interaction between PCD and oxidative stress Nguyen and Sutton (2009) and provide supporting evidence for our interpretation of the adverse effects of ROS accumulation on pollen viability and sterility.

The appropriate structure of anther and pistil is essential for successful pollination and fertilization, especially under stress conditions (Lord and Russell 2002). In our study, toluidine blue staining and degeneration assays of the anther and pistil under HS (Fig. 5 E, F) showed that structural alterations in the anther and pistil were significantly more pronounced in the susceptible variety (DH13) than in the tolerant one (SAFI5) when exposed to HS. It is well established that flower receptivity, particularly at the stigma, is critical for pollination, seed set, and overall plant performance. The stigma is the first part of the pistil to encounter pollen during pollination, initiating hydration and germination processes (Hiscock and Allen 2008). Our findings, along with analyses of O_2_^−^ content, accumulation sites, and microscopy of the anther and pistil, indicated that stigmas were more severely impacted by subsequent oxidative stress than pollen in both varieties (Fig. 5). This observation supports previous research highlighting the vulnerability of female reproductive organs to HS (Chen et al. 2021; Macova et al. 2022).

Stigmatic receptivity refers to the specific time during which the stigma creates an optimal environment for pollen germination (Sanzol et al. 2003). This aspect has significant biologic implications. In addition, the correlation between peroxidase activity and stigmatic receptivity indicates that receptive stigmas tend to accumulate higher levels of ROS, particularly hydrogen peroxide (H_2_O_2_) (McInnis et al. 2006; Hiscock and Allen 2008). Our findings suggest that the reduction in seed set of rapeseeds under HS may be linked to ROS accumulation, which corresponds with structural changes and damage observed in anthers and pistils. This is consistent with previous research indicating that plants respond to HS by activating various pathways, including ROS scavenging systems and signal transduction mechanisms (Larkindale et al. 2005; Fortunato et al. 2023). It has been proposed that there are common mechanisms governing both reproductive development and defense responses, including ROS induction, which could be crucial for understanding how plants react to HS (Burke and Chen 2015). Furthermore, it has been noted that ROS might have dual roles: acting as a positive signaling molecule triggering defense responses at low levels while causing stress damage and PCD at excessive levels (Dat et al. 2000; Petrov et al. 2015). Given the significant increase in mitochondria within pollen, leading to heightened respiration during pollen tube growth and development, an elevation in ROS levels during temperature stress is expected (Selinski and Scheibe 2014). Various functions associated with ROS production in plant mitochondria include redox/retrograde signaling, PCD, hormone action, and pathogen defense (reviewed in Huang et al. 2016).

Numerous studies have demonstrated that the scavenging capacity of ROS, as well as additional expression of antioxidant enzymes such as superoxide dismutase (SOD), catalase (CAT), and ascorbate peroxidase (APX) greatly contribute to plant adaptation, injury, and tolerance under adverse environmental conditions (Hasanuzzaman et al. 2014; Petrov et al. 2015; Chaki et al. 2020; Fortunato et al. 2023). However, little information is available regarding the interaction between HS-induced ROS content and diverse antioxidant enzyme activities in rapeseed reproductive organs. In our study, we observed significant effects of HS in catalase (CAT) and superoxide dismutase (SOD) activities but no significant difference in ascorbate peroxidase (APX) activity was noted in either anthers or pistils (Fig. 3 and 4). In addition, we found that the tolerant variety (SAFI5) exhibited higher superoxide dismutase (SOD) and catalase (CAT) activities compared to the sensitive one (DH15) under HS, while both showed a similar enzymatic response under control temperature conditions.

Among antioxidant enzymes, superoxide dismutase (SOD) serves as the first line of defense against the accumulation of ROS by catalyzing the dismutation of harmful superoxide radicals into hydrogen peroxide (H_2_O_2_), thus playing a crucial role in the antioxidant defense mechanism (Abreu and Cabelli 2010; Kayihan et al. 2012). Our findings revealed a significant impact of HS on both total superoxide dismutase (SOD) activity and its gene expression (Fig. 3), as the heat tolerant variety demonstrated markedly higher superoxide dismutase (SOD) activity in developing anthers and pistils compared to the sensitive variety. Similar increases in superoxide dismutase (SOD) activity have been observed in stress-tolerant genotypes under conditions of HS (Krishnan and Ramakrishnan 2011; Sharma et al. 2023) or salinity (Shalata et al. 2001). In contrast, ascorbate peroxidase (APX) activity in rapeseed anthers and pistils was less affected by HS during the reproductive phase when compared to catalase (CAT) and superoxide dismutase (SOD). These results are consistent with findings in rice (Zhao et al. 2018), which reported HS-induced changes in superoxide dismutase (SOD) activity within developing anthers. The role of catalase (CAT) involves converting H_2_O_2_ into water in an energy-efficient manner, which partially overlaps with ascorbate peroxidase (APX) function in non-photosynthetic tissues (Gill and Tuteja 2010). In non-photosynthetic plant tissues, excess H_2_O_2_ is primarily managed by catalase (CAT), which converts it to water (Foyer and Noctor 2000). Based on our observations regarding HS-induced changes in catalase (CAT) and superoxide dismutase (SOD) activities in anthers and pistils, it can be suggested that the scavenging role of ascorbate peroxidase (APX) may be compensated for or replaced by superoxide dismutase (SOD) and catalase (CAT) in response to elevated temperatures.

Conversely, catalase (CAT) is a vital antioxidant enzyme primarily found in peroxisomes (Mhamdi et al. 2010; 2012; Palma et al. 2020). Plant peroxisomes are organelles distinguished by a single membrane and a characteristic spherical shape, exhibiting a highly dynamic metabolism that participates in various essential physiologic functions (Corpas and Barroso 2015; Goto-Yamada et al. 2015; Oikawa et al. 2015; del Río und López-Huertas 2016). In addition to its involvement in ROS metabolism, research has revealed that plant peroxisomes harbor various molecules with significant signaling capabilities, such as nitric oxide (NO) and hydrogen sulfide (H_2_S), thereby broadening the functional scope of peroxisomes (Palma et al. 2020). Furthermore, there is substantial evidence of crosstalk between peroxisomes and other subcellular compartments, including mitochondria and plastids, which are closely interconnected at both biochemical and structural levels (Palma et al. 2006, 2023; Oikawa et al., 2019). Our findings indicate that HS caused notable changes in catalase (CAT) and superoxide dismutase (SOD) activity within the reproductive organs of rapeseed. Therefore, it can be proposed that both enzymatic systems are crucial for regulating ROS levels in rapeseed reproductive organs through the integration of regulatory mechanisms within mitochondria and peroxisomes.

Oxidative stress can be induced by various abiotic factors, such as HS, as noted by Mittler (2002). This condition leads to the production of ROS, which result in damaged proteins and cell death. Autophagy is one of several mechanisms that help proteome to be protected from damaging effects of HS. Autophagosomes serve as sites for the degradation and recycling of cellular materials and organelles. Our findings revealed an increase in autophagosome formation in both pistil and anther under HS condition. Notably, these structures were located at the top of the pistil and the bottom of the anther, where there was also an accumulation of superoxide and observable structural changes. These results suggest that autophagy plays a role in alleviating the harmful effects of ROS in the reproductive organs of rapeseed. Previous studies have documented the function of autophagy in removing protein aggregates in mutant lines of Arabidopsis and tomato (Zhou et al. 2013, 2014). Our experiment offers new insights into the role of autophagy in HS tolerance, specifically within the pistil and anther of *Brassica napus*.

Autophagy is a crucial process that facilitates the degradation of misfolded and denatured proteins produced because of HS (Su et al. 2020). A group of autophagy-related (ATG) proteins is involved in various stages of the autophagic process, with ubiquitin-like protein ATG8 playing a central role (Bu et al. 2020; Zhang et al., 2021). Our findings revealed that the expression of the *ATG8D* gene increased under HS in both reproductive organs of the two rapeseed varieties, indicating the formation of autophagosomes in both the pistil and anther (Figs. 6 and 7). Previous studies have established that ATG8 is a key protein in the autophagy process across several species, including Arabidopsis, rice, and tomato (Michaeli et al. 2014; Wang et al. 2015 and 2016; Fan et al. 2020). In addition, Sedaghatmehr et al. (2019) reported an interaction between ATG8 and HSPs, concluding that autophagy aids in the degradation of unfolded proteins and HSPs. Furthermore, temperatures reaching up to 30 °C have been shown to enhance autophagy in both anther cells and microspores in Arabidopsis. Mutants lacking functional *ATG* genes exhibited impaired pollen development and anther dehiscence, suggesting that autophagy plays a significant role in tapetum degeneration and pollen growth under HS condition (Dündar et al. 2019). The increase in *ATG18a* levels in heat-stressed microspores results from heat-induced endoplasmic reticulum (ER) stress, which activates ER-phagy. The subsequent transport of the endoplasmic reticulum (ER) to the vacuole for degradation relies on the presence of the ATG18a protein (Lohani et al. 2024).

The exocyst component EXO70B is critical for autophagosome formation and likely contributes to transport processes related to autophagy (Chanoca et al. 2015). It has also been demonstrated that EXO70B may be involved in autophagy triggered by specific stressors (Kulich et al. 2013). Our results (Fig. 7) indicate that the *EXO70B* gene is responsive to HS in reproductive organs, as its expression significantly increased in both pistil and anther of the heat-tolerant variety SAFI5. This suggests that precise regulation of autophagic activities may play a role in conferring HS tolerance in rapeseed.

Recent studies have indicated that NBR1-mediated selective autophagy plays a significant role in the degradation of damaged or denatured proteins produced by HS (Zhou et al. 2013, 2014; Jung et al. 2020) as well as other environmental stresses (Zhang and Chen 2020; Zhang and Ling 2023). The *nbr1* mutant exhibits reduced heat resistance and accumulates high levels of insoluble and excessively ubiquitinated proteins under HS, suggesting that the autophagy adaptor NBR1 is responsible for degrading the ubiquitinated protein aggregates formed due to stress (Zhou et al. 2013). NBR1 functions as a cargo receptor that binds to both ATG8 and ubiquitin, facilitating their transport to the vacuole (Michaeli et al. 2016). In addition, a strong association between NBR1 and catalase (CAT) has been observed in Arabidopsis under HS conditions (Zhou et al. 2014). Knockout lines of the *NBR1* gene displayed elevated levels of catalase (CAT) isoforms, indicating that NBR1 may play a role in the selective scavenging of catalases directly or as part of pexophagy. In this study, we found that the expression of the *NBR1* gene was upregulated under HS in the pistil and anther of the heat-tolerant variety, while no significant changes were detected in the sensitive variety (Fig. 7). This aligns with findings that suggest NBR1 is essential for the formation of autophagic vesicles induced by HS and acts as a receptor for aggrephagy, which is crucial for maintaining proteostasis under both normal and HS conditions (Jung et al. 2020). Furthermore, NBR1 has been implicated in the degradation of chloroplast proteins during HS in Arabidopsis (Zhang and Ling 2023; Wan et al. 2023). Overall, these findings suggest that the responses to HS in rapeseed reproductive organs are closely linked to autophagy regulation, particularly through the modulation of NBR1. These results align with those recently reported by Lohani et al. (2024), which highlighted changes in the expression of various *ATG* and *UPS-related* genes in pollen development subjected to HS.

In this study we made an attempt to have an in-depth analysis of the physiologic and genetic mechanisms related to HS responses in reproductive organs of rapeseed. Previous research on model organisms like Arabidopsis, as well as crops such as tomato, poplar, and rice, has demonstrated that the plants can endure various environmental stresses by utilizing autophagy to break down and recycle damaged proteins and organelles (reviewed in Li et al. 2023). Autophagy can be induced by abiotic stresses, and plants deficient in this process tend to be highly sensitive to environmental challenges. In addition, the role of ROS scavenging or signaling in enhancing tolerance to wide range of abiotic stresses including heat, drought and salinity is well documented (Larkindale et al. 2005; Fortunato et al. 2023). Thus, it is reasonable to hypothesize that both autophagy and reactive ROS management may play significant role in how rapeseed reproductive organs respond to heat or other stresses e.g., drought and salinity. As this experiment was conducted on two rapeseed varieties, future research exploring a wider variety of rapeseed cultivars could help to demonstrate the conservation of both mechanisms and to identify more genetic factors linked to HS tolerance, potentially revealing new traits advantageous for breeding programs. Moreover, applying functional genomics techniques will shed light on the specific contributions of superoxide dismutase (SOD), catalase (CAT), and other related genes in responding to HS, offering deeper insights into their operational mechanisms.

## Conclusion

**Fig. 8:**
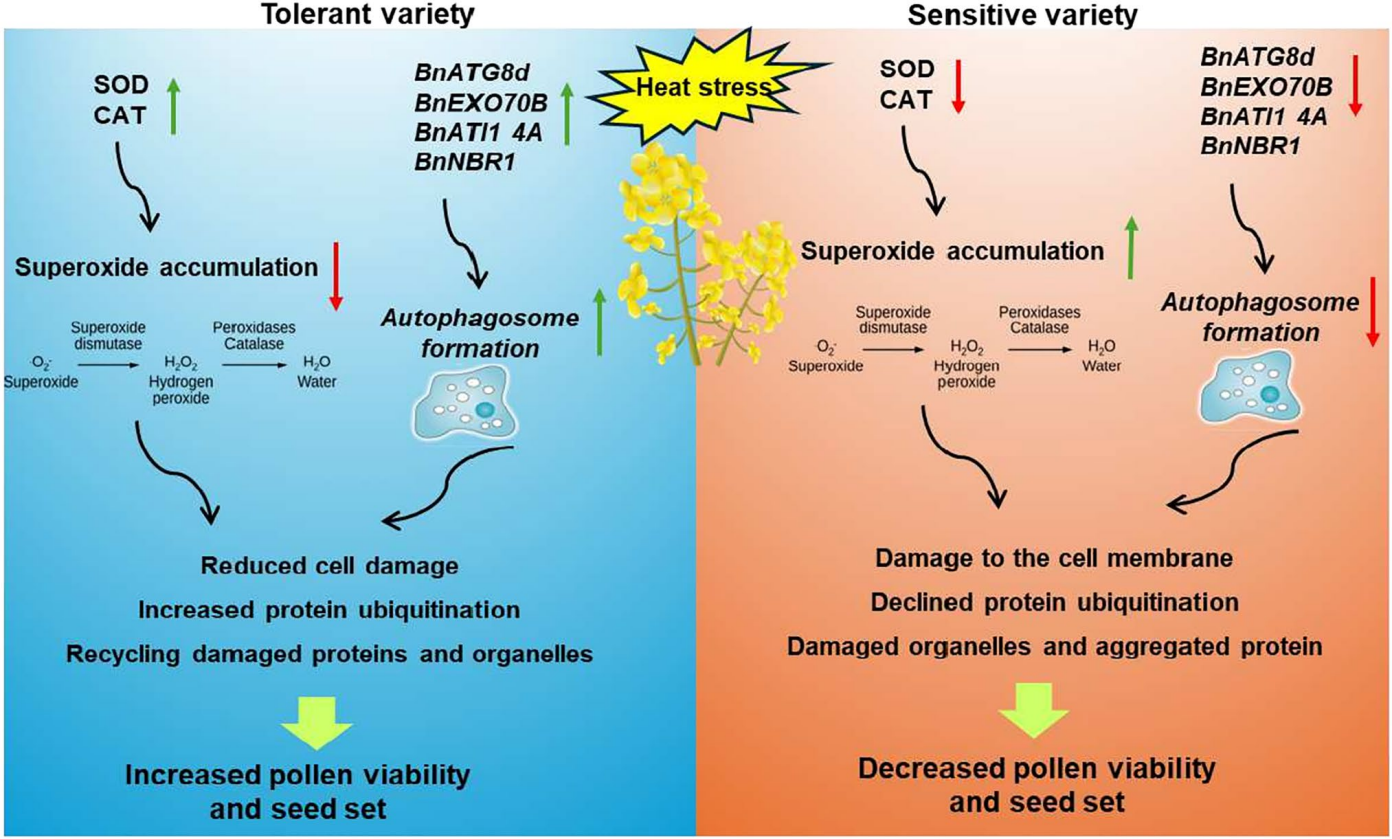
Antioxidant and autophagy mechanisms in response to heat stress in reproductive organs of rapeseed verities, DH13 and SAF15. Interaction of antioxidant and autophagy mechanisms lead to reduction of negative effects of superoxide and to recycle damaged proteins and organelles in pistil and anther

Heat stress during the reproductive stage poses a significant environmental challenge that can severely hinder the growth and productivity of rapeseed. This study highlights the essential roles played by antioxidant enzymes, particularly superoxide dismutase (SOD) and catalase (CAT), as well as autophagy in enhancing heat stress tolerance within the reproductive organs of rapeseed plants. The findings indicate that the heat-tolerant variety demonstrates elevated activity and expression levels of these antioxidant enzymes and associated genes, which are vital for detoxifying reactive oxygen species (ROS) and preserving cellular integrity under stressful conditions (Fig. 8).

These insights pave the way for developing innovative breeding and genetic engineering strategies aimed at producing rapeseed varieties with improved heat tolerance. By focusing on enhancing the expression of key antioxidant enzymes or autophagy genes breeders can create cultivars that not only withstand high temperatures but also maintain yield and quality in adverse climatic conditions.

## Supplementary Information

Below is the link to the electronic supplementary material.

Supplementary file1 (PPTX 499 KB)

## Data Availability

All data supporting the findings of this study are available within the paper or supplements. The plant materials used in this study are common rapeseed varieties of Iran, which are conveniently available in the country for university professors and researchers. The authors declare that all permissions and license were obtained.
