# Supplementary material for: Unraveling the role of autophagy and antioxidants in anther and pistil responses to heat stress in rapeseed (Brassica napus L.)
Source: Plant Cell Rep. 2025 Feb 7;44(2):51. doi: 10.1007/s00299-025-03437-6 (PMC11805782; doi:10.1007/s00299-025-03437-6)
Supplement: Supplementary file 1 — Supplementary file1 (PPTX 499 KB) [file 299_2025_3437_MOESM1_ESM.pptx]

## Slide 1
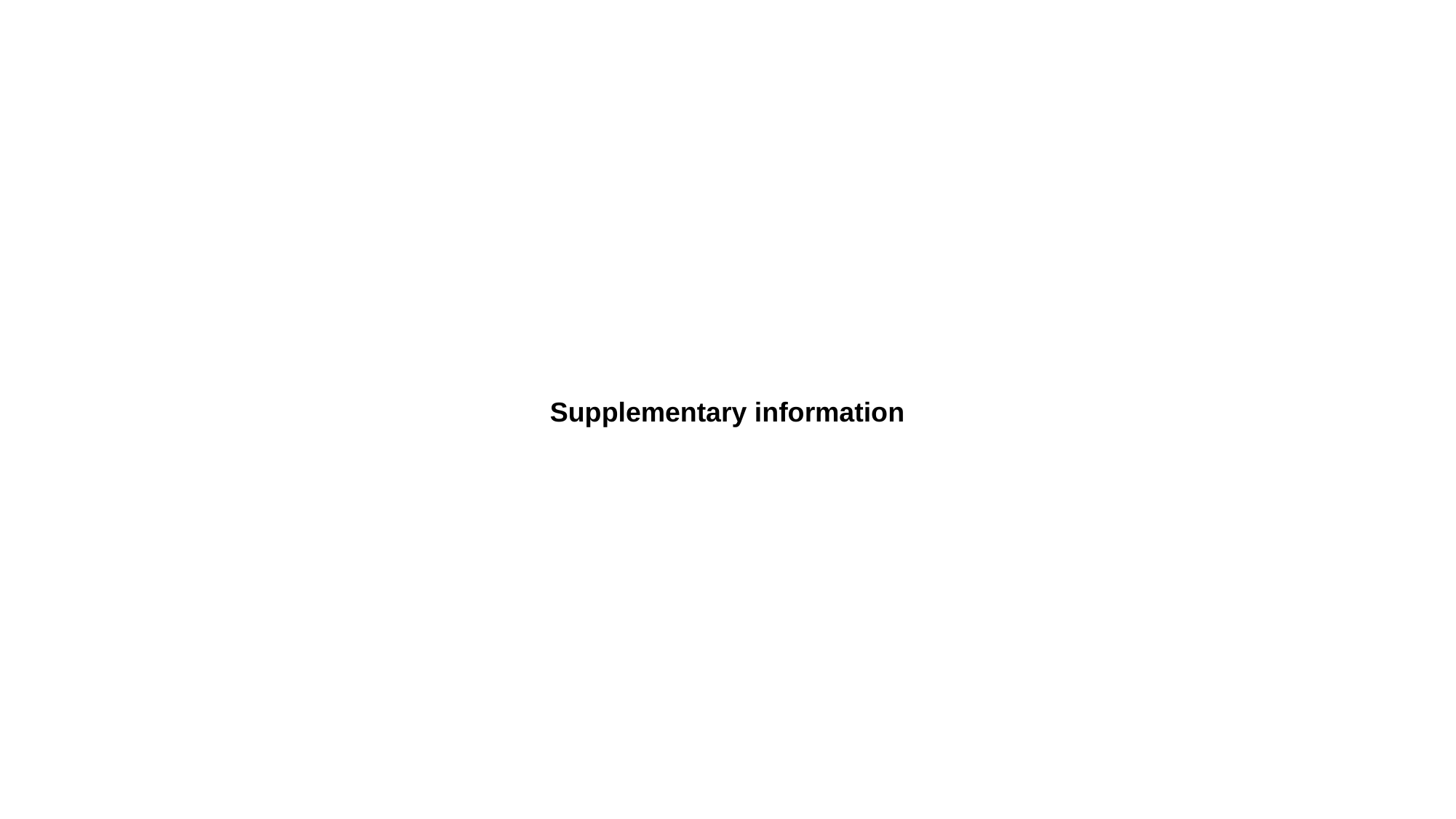

Supplementary information

## Slide 2
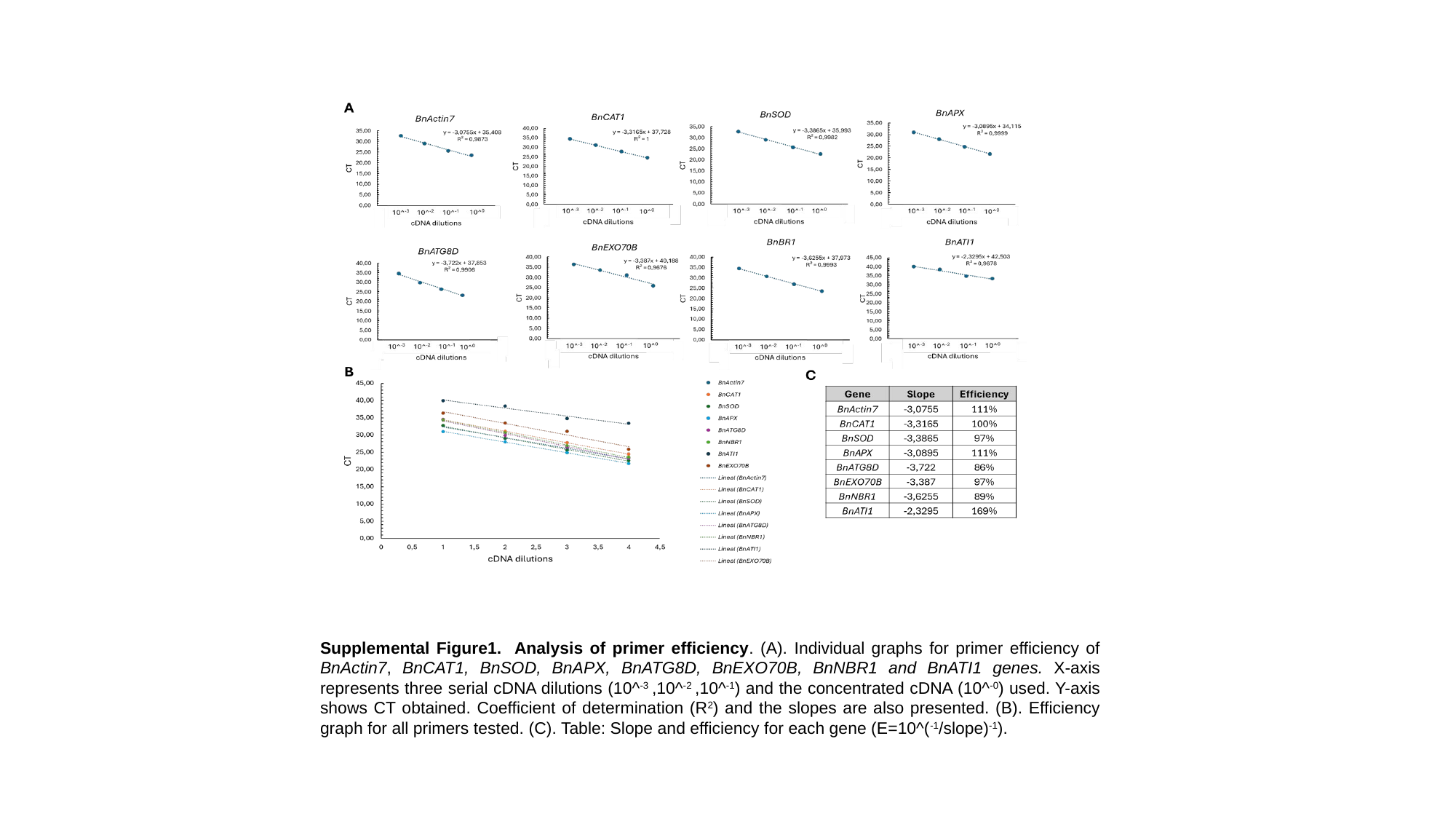

Supplemental Figure1. Analysis of primer efficiency. (A). Individual graphs for primer efficiency of BnActin7, BnCAT1, BnSOD, BnAPX, BnATG8D, BnEXO70B, BnNBR1 and BnATI1 genes. X-axis represents three serial cDNA dilutions (10^-3 ,10^-2 ,10^-1) and the concentrated cDNA (10^-0) used. Y-axis shows CT obtained. Coefficient of determination (R2) and the slopes are also presented. (B). Efficiency graph for all primers tested. (C). Table: Slope and efficiency for each gene (E=10^(-1/slope)-1).

## Slide 3
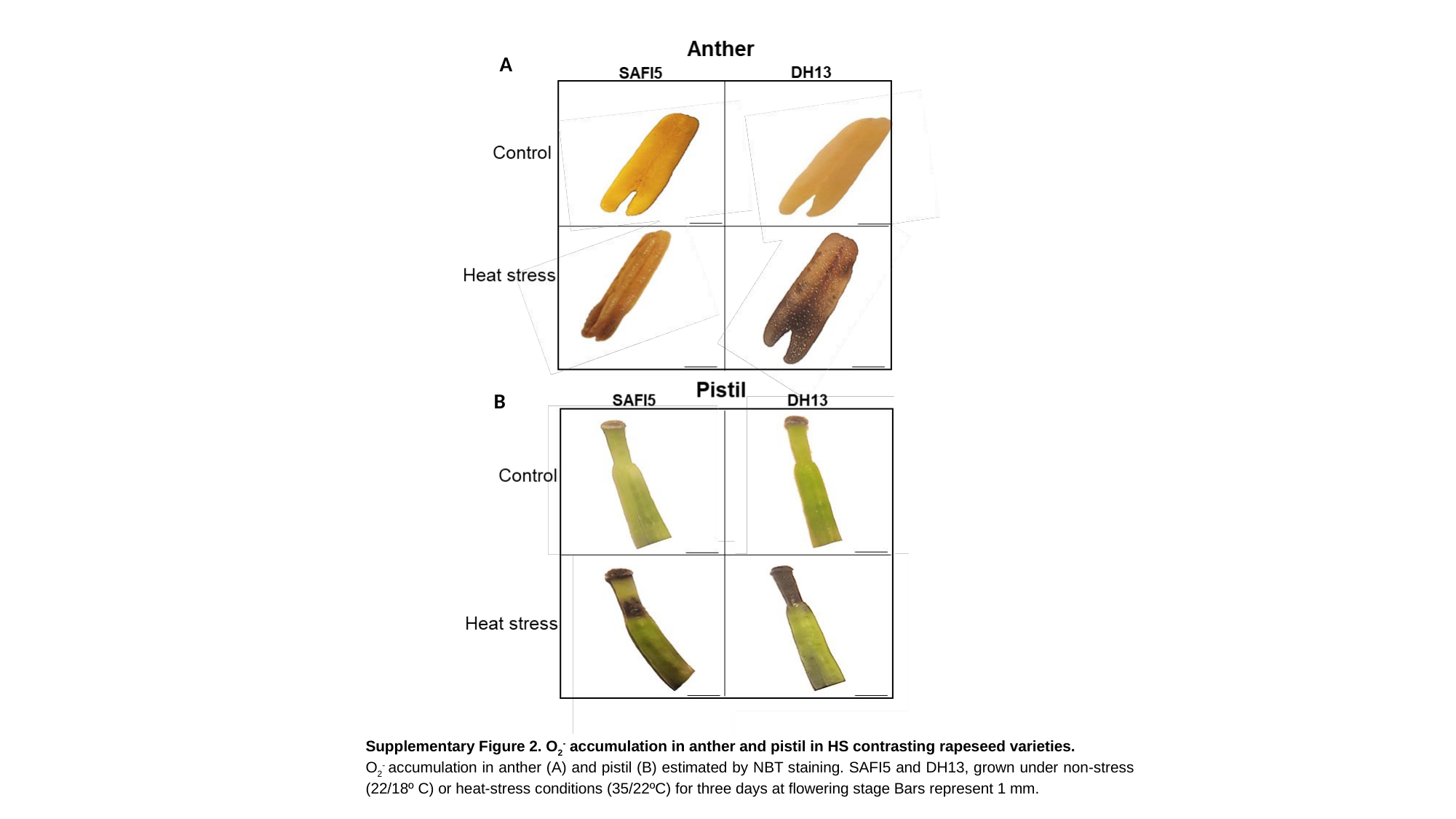

A
B
Supplementary Figure 2. O2- accumulation in anther and pistil in HS contrasting rapeseed varieties.
O2- accumulation in anther (A) and pistil (B) estimated by NBT staining. SAFI5 and DH13, grown under non-stress (22/18º C) or heat-stress conditions (35/22ºC) for three days at flowering stage Bars represent 1 mm.
